# Molecular Mapping and Transfer of Quantitative Trait Loci (QTL) for Sheath Blight Resistance from Wild Rice *Oryza nivara* to Cultivated Rice (*Oryza sativa* L.)

**DOI:** 10.3390/genes15070919

**Published:** 2024-07-14

**Authors:** Kumari Neelam, Sumit Kumar Aggarwal, Saundarya Kumari, Kishor Kumar, Amandeep Kaur, Ankita Babbar, Jagjeet Singh Lore, Rupinder Kaur, Renu Khanna, Yogesh Vikal, Kuldeep Singh

**Affiliations:** 1 School of Agricultural Biotechnology, Punjab Agricultural University, Ludhiana 141004, India; 2 Department of Plant Pathology, Punjab Agricultural University, Ludhiana 141004, India; 3 ICAR—Indian Institute of Maize Research, PAU Campus, Ludhiana 141004, India; 4 Division of Agricultural Biotechnology, Ramakrishna Mission Vivekananda Educational and Research Institute, Narendrapur Campus, Kolkata 700103, India; 5 Department of Plant Breeding and Genetics, Punjab Agricultural University, Ludhiana 141004, India; 6 International Crops Research Institute for the Semi-Arid Tropics (ICRISAT), Patancheru 502324, India

**Keywords:** sheath blight disease, *O. nivara*, *R. solani*, QTL mapping, SSR markers, linkage map, pre-breeding

## Abstract

Sheath blight (ShB) is the most serious disease of rice (*Oryza sativa* L.), caused by the soil-borne fungus *Rhizoctonia solani* Kühn (*R. solani*). It poses a significant threat to global rice productivity, resulting in approximately 50% annual yield loss. Managing ShB is particularly challenging due to the broad host range of the pathogen, its necrotrophic nature, the emergence of new races, and the limited availability of highly resistant germplasm. In this study, we conducted QTL mapping using an F_2_ population derived from a cross between a partially resistant accession (IRGC81941A) of *Oryza nivara* and the susceptible rice cultivar Punjab rice 121 (PR121). Our analysis identified 29 QTLs for ShB resistance, collectively explaining a phenotypic variance ranging from 4.70 to 48.05%. Notably, a cluster of four QTLs (*qRLH1.1*, *qRLH1.2*, *qRLH1.5*, and *qRLH1.8*) on chromosome 1 consistently exhibit a resistant response against *R. solani.* These QTLs span from 0.096 to 420.1 Kb on the rice reference genome and contain several important genes, including Ser/Thr protein kinase, auxin-responsive protein, protease inhibitor/seed storage/LTP family protein, MLO domain-containing protein, disease-responsive protein, thaumatin-like protein, Avr9/Cf9-eliciting protein, and various transcription factors. Additionally, simple sequence repeats (SSR) markers RM212 and RM246 linked to these QTLs effectively distinguish resistant and susceptible rice cultivars, showing great promise for marker-assisted selection programs. Furthermore, our study identified pre-breeding lines in the advanced backcrossed population that exhibited superior agronomic traits and sheath blight resistance compared to the recurrent parent. These promising lines hold significant potential for enhancing the sheath blight resistance in elite cultivars through targeted improvement efforts.

## 1. Introduction

Sheath blight (ShB) is caused by a soil-borne necrotrophic fungus *Rhizoctonia solani* Kühn (*R. solani*). It is the most serious disease of rice (*O. sativa* L.) that threatens its productivity and quality [1,2]. Globally, it can cause up to 50% yield loss under severe conditions [3,4]. The ShB pathogen survives in soil and water as sclerotia and remains viable for up to three years as mycelium [5]. It infects the sheath of rice plants above the waterline, spreading upwards to create lesions that soften and lodge stems, inhibit grain filling, reduce leaf area, and infect tillers, ultimately causing significant yield loss [6,7]. Dense planting, warm and humid weather, the application of high doses of nitrogen fertilizers, and adaptation of susceptible semi-dwarf varieties exacerbate the disease [8]. Controlling ShB is challenging due to the resurgence of resistant pathogen strains, the broad host range, and the lack of highly resistant germplasm [9]. Chemical fungicides represent a key strategy in controlling this disease; however, their overuse results in resistant strains, increased expenses, and poses risks to both the environment and human health [10,11]. Breeding cultivars resistant to sheath blight is a highly economical and effective strategy, requiring the identification of resistant donors from diverse sources. Researchers have identified some partially resistant donors from cultivated germplasm and a few landraces, but effective, heritable resistance from wild species remains limited [9,12].

ShB resistance is a complex trait governed by the combination of major and minor genes [9,13,14]. Li et al. [15] identified sheath blight resistance QTLs for the first time using restriction fragment length polymorphism (RFLP) markers in the F4 population derived from the Lemont/Teqing cross. Subsequently, multiple QTLs for sheath blight resistance, scattered across the 12 rice chromosomes have been mapped [16,17,18,19,20,21,22,23,24]. Most of them are reported as minor QTLs, except for a few major QTLs including *qShB9-2* and *qSBR11-1* [20,25]. A few loci (*qSB-11LE* and *qSB-9TQ*) have been fine-mapped, delineated into short genomic regions with a small number of candidate genes [22,23]. The GWAS studies reliably identified significant loci associated with ShB resistance over several years [26,27,28,29]. Despite the identification of candidate genes in many cases, their role in sheath blight resistance has not been fully verified. Further research and validation are needed to understand ShB resistance mechanisms in rice and optimize crop breeding strategies to combat this destructive disease.

The wild species germplasm of rice plays a crucial role in enriching the genetic diversity of modern rice cultivars [30,31]. In our previous report, a wild accession of *O*. *nivara* (accession no. IRGC81941A) was identified as a promising donor showing partial resistance to *R. solani* [32]. The present investigation aims to map ShB resistance QTLs using an interspecific population derived from a cross between moderately resistant accession of *O. nivara* acc. IRGC81941A and highly susceptible cultivar PR121. The research successfully developed pre-breeding lines with superior agronomic traits and high tolerance to sheath blight disease, along with linked markers for marker-assisted selection (MAS).

## 2. Materials and Methods

### 2.1. Plant Materials and Population Development

Punjab Agricultural University (PAU), India, maintains an active collection of around 1600 wild species germplasm, sourced from the ICAR-National Rice Research Institute (ICAR-NRRI) in Cuttack, India, and the International Rice Research Institute (IRRI) in Los Baños, Philippines. A subset of approximately 400 accessions of *O. nivara* was screened for resistance against a highly virulent strain of *R. solani* (RS-1) in the Punjab region. Out of these, 67 accessions of *O. nivara* exhibited moderately resistant responses against ShB for two years of screening [32]. One partially resistant accession, *O*. *nivara* acc. IRGC81941A, consistently showed a resistant response (disease score of 3.0 on a 0–9 scale) with the least relative lesion height (22.80%). This accession was selected for genetic mapping of ShB-resistant QTLs. It was crossed as a male parent to PR121, a high-yielding, semi-dwarf mega variety highly susceptible to *R. solani*. The F_1_ hybrid was self-pollinated to produce F_2_, F_2_:_3_, F_3_:_4_, and F_4_:_5_ populations. The F_2_ and F_2_:_3_ populations and their means were used for QTL mapping studies.

### 2.2. Development of Backcross Populations 

**Figure 1:**
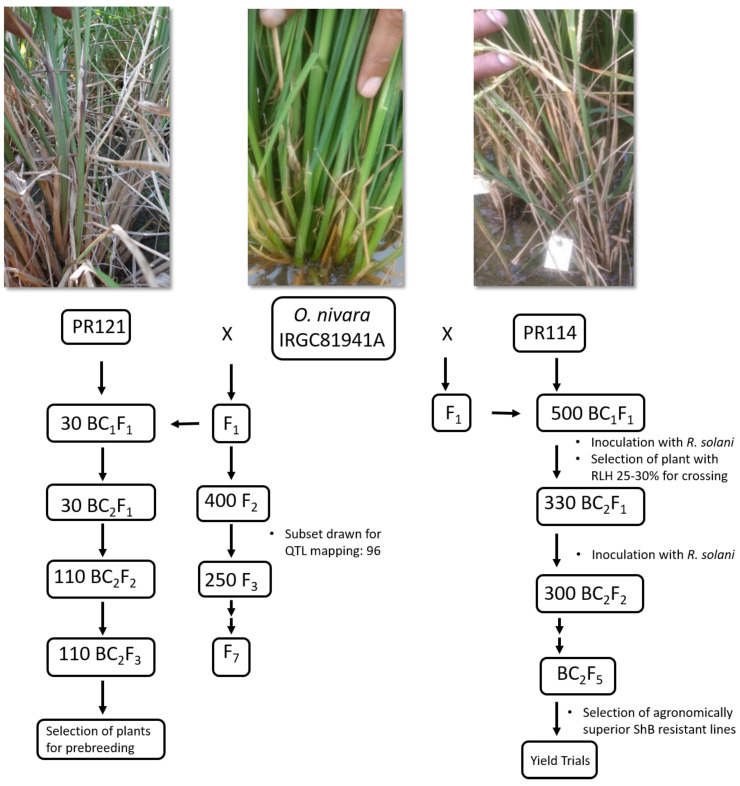
Schematic representation of the development of mapping and backcross populations in the genetic background of susceptible cultivars.

To introgress the ShB resistance QTLs, the F_1_ hybrids were backcrossed with two different susceptible cultivars, PR121 and PR114. Approximately 500 BC_1_F_1_ plants were developed by crossing PR114 with the tolerant parent *O. nivara* acc. IRGC81941A. The BC_1_F_1_ plants were inoculated, and those with RLH between 25 to 30 percent were backcrossed to generate BC_2_F_1_. Further, 330 BC_2_F_1_ plants from four different BC_1_F_1_ progenies (progeny no. 6052, 6053, 6054, and 6055) were inoculated and advanced to BC_2_F_2_. Four BC_2_F_2_ progenies (6645-6, 6645-12, 6646-13, and 6646-16) were agronomically good with small awns, and optimum plant heights ranging from 70–90 cm, were selected. In contrast, some BC_2_F_2_ progenies were discarded due to late flowering, big awns, and tall stature. Approximately 300 BC_2_F_2_ plants and corresponding three plants of each BC_2_F_3_ progenies (1547, 1554, and 1556) were inoculated, and data for plant height, lesion length, disease score, and days to flowering were recorded. The BC_2_F_3_ progeny 1547 was developed in the genetic background of highly susceptible parent PR121, whereas 1554 and 1556 were developed in the genetic background of PR114. A graphical representation showing the development of mapping populations is provided in Figure 1.

### 2.3. Preparation of Pure Culture and Disease Assessment

**Figure 2:**
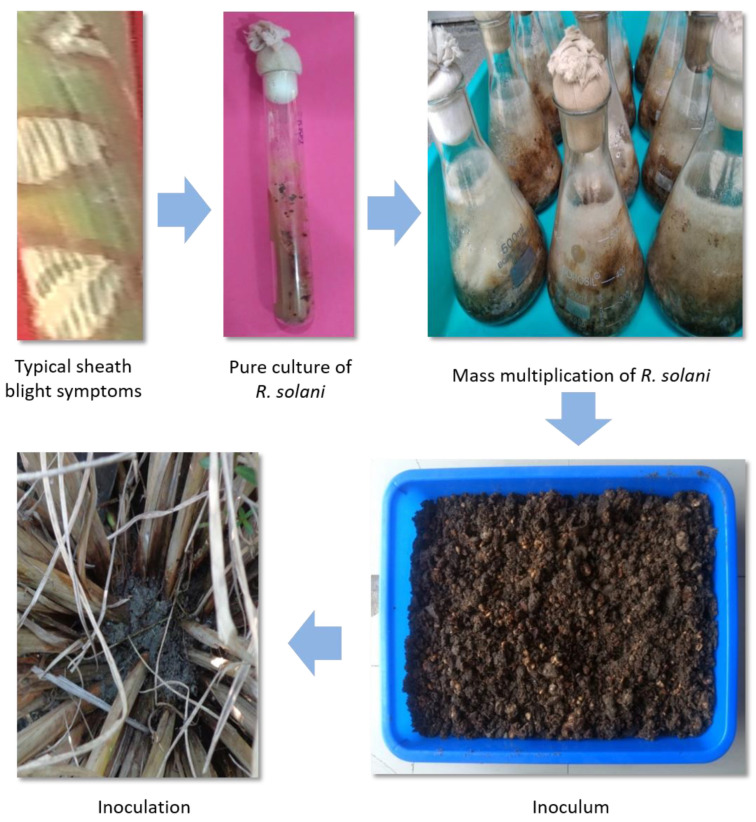
Isolation of pure culture from the highly virulent strain RS-1 of *R. solani* and preparation of inoculum based on maize meal sand medium. Five grams of inoculum were inoculated on the hill of each rice plant for evaluation.

A highly virulent strain of *R. solani* (RS-1) was isolated from a local rice cultivar PR116 from the experimental field of PAU, Ludhiana [33]. The infected leaf sheath was thoroughly washed under running tap water and cut into small pieces. The small pieces of leaf sheath were surface sterilized with 0.1% mercuric chloride for 1 min in a beaker and washed three times with autoclaved distilled water. The surface-sterilized sheath pieces were inoculated onto potato dextrose agar medium (PDA). The plates were incubated at 26 ± 2 °C for 5–7 days under a BOD incubator. The maize meal–sand (1:3) medium supplemented with 20 g sucrose was used for mass multiplication [33]. For screening, five grams of inoculums were placed in the center of a hill of each F_2_ individual (Figure 2). The observation was recorded by measuring the lesion length 14 days after inoculation (DAI). The disease reaction, based on lesion length (cm), was classified according to the scale given in the Standard evaluation system of rice. The relative lesion height is the average vertical height of the uppermost lesion on a leaf or sheath expressed as a percentage of the average plant height. Lesion lengths limited up to 20% plant height (disease score 0–1) was classified as resistant, between 20–30% (disease score 3) as moderately resistant, 31–45% (disease score 5) as moderately susceptible, 46–65% (diseases score 7) and greater than 65% disease scored as 9. Plant height (PH) was also measured from bottom to top leaf. Relative lesion height (RLH) was calculated according to the formula given by [34].

RLH (%) = (Lesion height/Plant height) × 100

For the screening of F_2:3_ populations, each F_2_ family was planted in a progeny row comprising ten plants. The plants were inoculated with *R. solani*, and phenotypic data for disease variables were recorded after 14 days of inoculation. The mean of the ten plants was used in further analysis. Descriptive statistics, density plots, and correlation studies were performed in the ggplot2 package of R software version 4.1.2 (https://ggplot2.tidyverse.org, accessed on 4 October 2021). 

### 2.4. DNA Extraction and SNP Genotyping

A large-scale DNA extraction protocol was used to isolate high-molecular-weight DNA from 25 to 30 days old field-grown F_2_ plants using CTAB (cetyl trimethyl ammonium bromide) method. DNA concentration was quantified by spectrophotometer (Eppendorf Biophotometer). Integrity and quality of genomic DNA were performed by separating the DNA on 0.8% agarose gel. All the DNA samples were normalized to equal concentration by adding 1X Tris EDTA buffer. The genotyping of the F_2_ population and parents was outsourced to AgriGenome using double-digest restriction site-associated DNA sequencing (ddRAD-seq). For genotyping, DNA samples from each F_2_ plant and the parents (1000 ng) were digested with two restriction enzymes *SphI* and *MlucI* (Non-methylation sensitive) used for ddRAD sequencing. The sequencing was performed using the Illumina HiSeq 2000/2500 sequencing platform. Single-nucleotide polymorphisms (SNPs) were identified by aligning the separate fastq files of reads with the Nipponbare reference IRGSP 1.0 SNPs using Bowtie2 [35]. The alignments were transformed to the SAM (sequence alignment map) format and combined into a single BAM (binary file of SAM) using SAMtools 0.1.18. SNPs were called using the genome analysis toolkit (GATK) using the BAM file. Raw SNP data were filtered using the threshold for SNP call rate ≥ 0.7 and minor allele frequency (MAF) ≥ 0.05.

SNP markers with <10% missing data were used for the construction of a genetic map. Segregation distortion in marker data was assessed through the Chi-square test. The SNP markers with a Chi-square value >10 were considered distorted and excluded from the linkage analysis. RECORD 2.0 (REcombination Counting and ORDering) software was used for ordering the markers on the linkage groups (LGs) [36]. The ordering of markers was performed using default functions with the Kosambi mapping function and a 30% recombination rate. In the case of multiple SNPs showing similar segregation patterns, all the markers were placed in the same genetic bin and only one representative marker was used for the final map construction. The linkage map was drawn using MapChart 2.0 software [37]. 

### 2.5. QTL Mapping

The QTL analysis was performed for ShB and related traits using a composite interval mapping (CIM) function with a window size of 10 cM in Windows QTL Cartographer 2.5 software (http://statgen.ncsu.edu/qtlcart/WQTLCart.htm, accessed on 10 July 2024). The threshold logarithm of the odds (LOD) scores was determined using 1000 permutations [38]. The CIM model was selected based on markers used as cofactors by forward and backward regression analyses with a walking speed of 10 cM. The observed phenotypic variance (*R*^2^) exhibited by the QTL was estimated by the CIM model.

### 2.6. Statistical Analysis

#### 2.6.1. Phenotypic Data Analysis

The replicated data for disease variables were recorded in each generation, 14 days after inoculation, following the standard scale. For screening F_2:3_ populations, phenotypic data from ten plants per F_2_ family were averaged for further analysis. Descriptive statistics, density plots, and correlation studies were conducted using the ggplot2 package in R 4.1.2 software (https://ggplot2.tidyverse.org, accessed on 10 July 2024). 

#### 2.6.2. SNP Data Analysis

For SNP analysis, SNPs were identified by aligning fastq files with the Nipponbare reference IRGSP 1.0 using Bowtie2. Alignments were converted to SAM format and merged into a BAM file with SAMtools. SNPs were called using GATK and filtered for a call rate ≥ 0.7 and MAF ≥ 0.05. SNPs were identified and filtered based on call rate and minor allele frequency. Segregation distortion were analyzed using Chi-square test. 

#### 2.6.3. Linkage and QTL Mapping

The SNP-based linkage map was constructed using RECORD 2.0 package [36]. The linkage maps were drawn using MapChart 2.32 program [37]. QTL mapping was performed in QTL Cartographer 2.5 (http://statgen.ncsu.edu/qtlcart/WQTLCart.htm, accessed on 10 July 2024) using composite interval mapping (CIM) model. The QTLs were detected threshold of 3.0 LOD with 1000 permutations and 10 cM of walking speed. 

### 2.7. Identification of the Linked Marker with Shb Resistance

To pinpoint the linked SSR markers for marker-assisted selection (MAS), we employed nearly 100 SSR markers within the identified QTLs region on the chromosomes 1, 3, and 11. The marker intervals Chr1.35880614-Chr1.38966610, Chr3.2694761-Chr3.3801316, Chr11.26053892-Chr11.28970389 on chromosome 1, 3, and 11, respectively, were choosen on the basis of colocalization of the identified QTLs for at least two contributing traits along with the consistency over the years and also on the mean data. The QTLs identified in our study were also colocalized with previously mapped ShB-resistant QTLs with a major effect in different populations suggesting that these genetic regions play a crucial role in conferring resistance to the ShB disease. We utilized these markers in both the RILs and backcross populations to facilitate the identification of SSR markers linked to the target traits for MAS. The SSR markers were amplified using a thermal cycler (Eppendorf Master Cycler, Hamburg, Germany) in a set of 25 μL PCR reactions. PCR products were separated in 2.5% agarose gel. The amplicons were scored by comparing with the size of alleles corresponding to the parental alleles.

### 2.8. Identification of Candidate Genes 

The coordinates of the flanking SNPs linked with the QTL were searched in the rice genome annotation project database (http://rice.plantbiology.msu.edu/, accessed on 10 July 2024) to determine the physical positions, size of the QTLs, and the number of open reading frames (ORFs). The putative candidate genes underlying the QTL were retrieved and analyzed. 

## 3. Results

### 3.1. Frequency Distribution of ShB Disease in the F_2_ and F_2:3_ Populations

**Figure 3:**
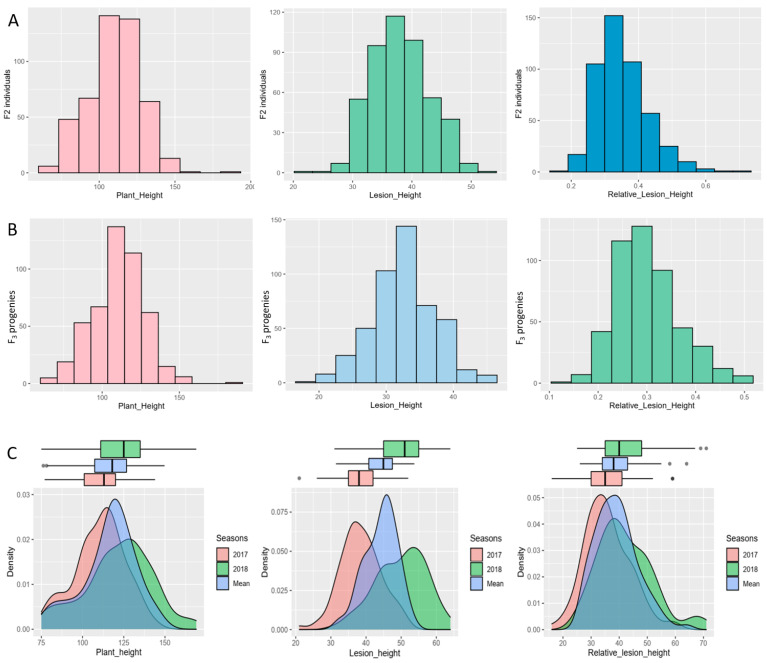
Distribution of plant height (PH), lesion height (LH), and relative lesion height (RLH) in different mapping populations. (**A**) Histogram showing the distribution of PH, LH, and RLH in the F_2_ population during the year 2017. (**B**) Histogram showing the distribution of PH, LH, and RLH in F_2:3_ populations during the year 2018. (**C**) Density plot representing frequency distribution of PH, LH, and RLH in F_2_, F_2:3,_ and their mean.

The resistant parent *O. nivara* acc. IRGC81941A and the highly susceptible recipient parent PR121 showed contrasting responses to *R. solani* during the five years of screening. *O. nivara* acc. IRGC81941A consistently showed a disease score of 3 on a 0–9 scale, with a mean RLH of 19–24% against a highly virulent strain of *R. solani* over the period from 2017 to 2021. The recipient parent PR121 and PR114 consistently displayed a score of 9 with a mean RLH of 62–75% over five years of screening against *R. solani*. Phenotypic evaluation for the three ShB-related traits, namely LH, RLH, and PH, was performed on 479 F_2_ individuals with their parental lines in the year 2017. The phenotypic data in 2017 ranged from 21 to 52 cm for LH, from 65 to 185 cm for PH, and from 0.15 to 0.70% for RLH, and. Continuous variations with transgressive segregation was observed in the F_2_ populations for LH, RLH, and PH (Figure 3A). The Shapiro–Wilk (*w*) normality test indicated that the phenotypic data in the F_2_ population were normally distributed (Table S1). As the population is derived from the wild species of rice, a number of plants did not set the seeds and we are left with only 241 plants. A total of 241 F_2:3_ families (10 plants each) were evaluated in the year 2018 against *R. solani* (Table S2). The phenotypic data for three traits were slightly skewed towards the recipient parent (Figure 3B). The average of F_2_ and F_2:3_ was more stabilized and exhibited a normal distribution (Figure 3C). Therefore, the mapping was conducted using mean data as well as F_2_ and F_2:3_ populations. 

The different disease variables measured in the years 2017 and 2018 and their means were significantly (*p* < 0.05) correlated (Figure S1). A strong positive correlation was observed between LH and RLH in 2017 (0.74), and 2018 (0.54), indicating that these two traits were highly associated with each other under different environmental conditions. PH showed a significantly negative correlation with LH and RLH in 2017 and 2018 and their means. RLH was negatively correlated to PH, with a correlation coefficient of −0.75 in 2017 and −0.77 in 2018, whereas LH displayed a negative correlation with PH in 2017 (−0.15), and a poor correlation in 2018 (0.08). 

### 3.2. Identification of Promising Lines in the BC_2_F_2_ and BC_2_F_3_ Populations 

**Figure 4:**
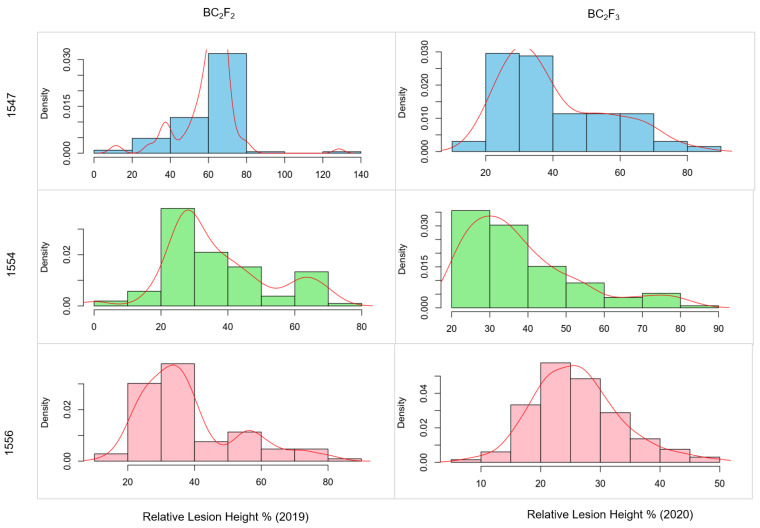
Frequency distribution of relative lesion height in three backcross populations (1547, 1554, and 1556) in the years 2019 and 2020. The red line represents the density curve.

Significant variations were observed for three traits, PH, LH, and RLH, in the BC_2_F_2_ and BC_2_F_3_ populations. BC_2_F_2_ plants with a resistance score of 3–5 and RLH < 28% along with desirable agronomic traits were selected for the development of prebreeding lines. These progenies were self-pollinated as BC_2_F_3_ progenies (1547, 1554, and 1556) and evaluated in 2020. The frequency distribution of RLH for three BC_2_F_2_ populations and their corresponding BC_2_F_3_ progenies is represented in Figure 4. The phenotypic distribution of RLH in the BC_2_F_2_ population was nearly symmetrical and bell-shaped. In contrast, the BC_2_F_3_ progenies generally skewed towards the resistant parent, except for population 1556, which exhibited a symmetrical distribution for the trait. A total of 13, 30 and 6 individuals from the progenies 1554, 1556, and 1547, respectively, were selected with a disease score of 3.0 and superior agronomic traits. These promising resistant BC_2_F_3_ lines exhibit substantial variation in major agronomic traits such as plant height, small awn, early maturity, without any significant reduction in the yield (Table S3). Transgressive segregation for plant height, earliness, short awns, and grain number indicates the contribution of beneficial and yield-enhancing alleles from wild species. The superior ShB-resistant lines showing desirable agronomic traits could be excellent prebreeding germplasm resources for broadening the genetic base of the cultivated gene pool.

### 3.3. Genotyping of the F_2_ Population Using ddRAD-seq 

A total of 3,32,663 SNP markers were obtained with ddRAD sequencing. SNP markers with indels or heterozygote SNPs in either of the parents were removed, and only SNPs in the homozygous state (AA, CC, TT, and GG) were used for scoring the polymorphism between *O. sativa* cv. PR121 and *O. nivara* accession IRGC81941A. With this approach, we were left with 51,320 polymorphic SNPs between the parents for further linkage analysis. After filtering significantly distorted markers (χ^2^ ≥ 10) and those with missing values ≥ 10%, a total of 1971 markers were retained for genetic map construction. The SNP markers with distorted segregation were analyzed using the Chi-square test. A total of 1971 markers were thus used for the construction of the linkage map using RECORD (Table S4). 

### 3.4. Linkage and QTL Mapping 

Based on an SNP-based linkage map with 1,971 SNP markers and phenotypic data, we conducted QTL analysis using composite interval mapping in QTL cartographer 2.5 software. A total of 29 QTLs were detected for sheath blight resistance and component traits using composite interval mapping. These QTLs were located on all the rice chromosomes except 5, 9, 10, and 12, and the total phenotypic variance explained (PVE) ranging from 4.70 to 48.05% (Table 1). Favorable alleles from 13 QTLs were inherited from the susceptible cultivars PR121, while 16 QTLs were inherited from partially resistant parent *O. nivara* acc.81941A. Among them, 11 QTLs were detected on chromosome 1. Five loci on chromosome 1 and four loci on chromosome 3 were mapped to the nearly same location and stable across the years 2017 and 2018 and the mean data, whereas other loci were mapped at a different position or were undetected under different environmental conditions. A total of eight QTLs were identified for PH, including four in the year 2017, two each in the year 2018, and two in the mean data. They were located on chromosomes 1, 2, 4, 6, and 8 and explaining 0.5 to 37.69% phenotypic variance. Among them, six QTLs were major, explaining >10% phenotypic variance, while two were minor QTLs. A major QTLs located on chromosome 1 was consistently detected in all the environments and the mean, exhibiting phenotypic variance ranging between 17.3 to 37.69%. This QTL was colocalized near the QTL controlling RLH in all environments and the mean. Eight QTLs were identified for LH, including five in the year 2017, one in 2018, and two in the mean, with R^2^ ranging from 4.82 to 23.08%. Thirteen QTLs were detected for RLH, four each in the years 2017 and 2018 and five in the mean data, and R^2^ ranged from 4.70–48.05%.

### 3.5. Identification of the Linked Markers for MAS 

**Figure 5:**
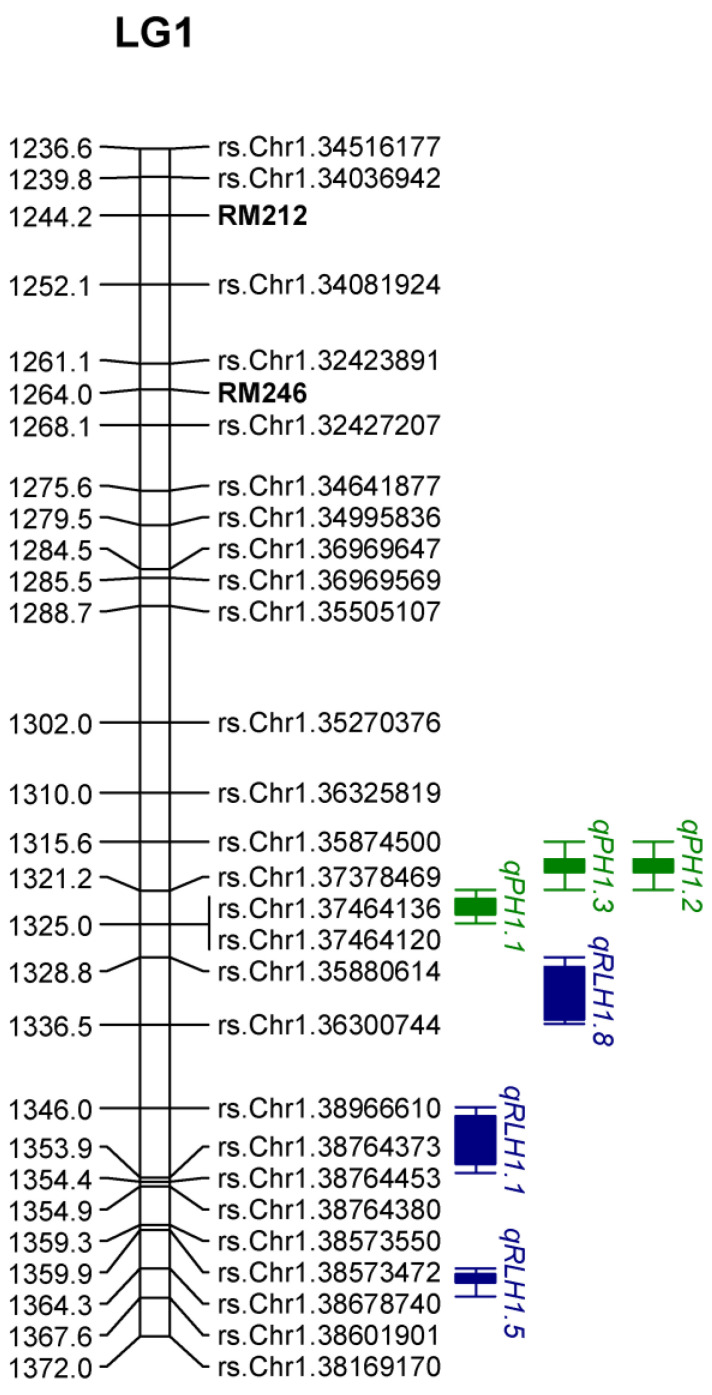
Location of stable QTL linked to SNP markers on the partial linkage map of chromosome 1. SSR markers are represented in bold.

Loci mapped on chromosomes 1, 3, and 11 were consistently detected across multiple generations. Out of 100 markers, 5 on chromosome 1 and 7 each on chromosomes 3 and 11 were found to be polymorphic between the parents (Figure S2). These markers were used on the F_2_ population to identify the introgressed QTLs conferring resistance to ShB. Of the 5 markers on chromosome 1, RM212 and RM246 showed normal Mendelian segregation and were included in the linkage map (Figure 5). The remaining markers exhibited distorted segregation in the F_2_ populations and were discarded. The SSR marker RM246 was found to be closely linked to the cluster of QTLs for RLH and is of utmost importance for MAS.

**Figure 6:**
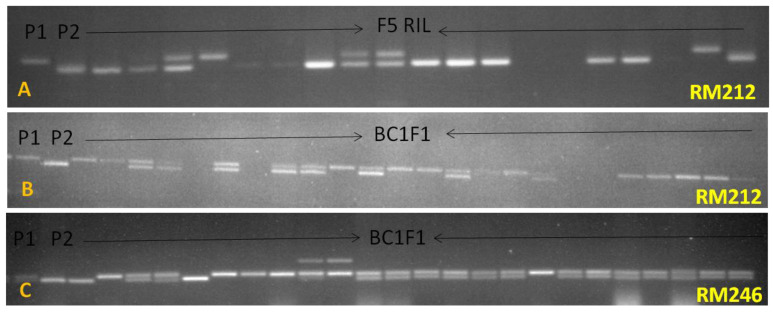
Agarose gel showing amplification of F_5_ RIL and BC_1_F_1_ population using SSR markers RM212 and RM246. (**A**,**B**) PCR profiling of F_5_ RIL and BC_1_F_1_ population using RM212, (**C**) Genotyping of BC_1_F_1_ population using RM246. P1 and P2 represent parental line PR121 and *O. nivara* acc. IR81941A, respectively. The presence of heterozygotes indicates the introgression of alleles from both parents.

To validate the linked markers, we applied them to the previously mentioned backcross populations and RILs. The backcross population derived from the progenies of 1547 (PR121/*O. nivara* IR81941A)//2*PR121) and RILs showed the introgression of chromosome 1 with the markers RM246 and RM212 (Figure 6). These lines displayed an RLH percentage between 19 and 30 percent when challenged with the pathogen. We were unable to detect the introgression of chromosomes 1, 3, and 11 with the SSR markers in the progenies from the other two crosses. This is likely due to the presence of small alien introgressions, which require further genotyping with a SNP platform for effective tracing and analysis.

Additionally, the SSR markers RM212 and RM246 were successfully amplified across various non-basmati and basmati rice cultivars and aromatic germplasm. Notably, the *O. nivara* allele could distinguish between susceptible and resistant lines, as most rice cultivars showed amplification patterns consistent with the susceptible variety PR114 (Figure S3). Interestingly, both traditional basmati and aromatic rice germplasm exhibited different alleles compared to those present in both parents. It is worth noting that sheath blight is not a common disease in basmati and aromatic germplasm. These findings highlight the high potential of this marker in marker-assisted selection (MAS) programs aimed at enhancing sheath blight disease resistance and ultimately improving rice crops.

### 3.6. Identification of Candidate Genes Underlying ShB Resistance QTL

The rice reference genome was searched using the coordinates of linked SNP markers associated with the QTLs. Stable QTLs *qRLH1.1* spanned at 202 kb genomic region, *qRLH1.2* in 0.096 kb, *qRLH1.5* in 76.84 kb, and *qRLH1.8* in 420.1 kb region harboring 35, 1, 10, and 67 candidate genes, respectively. The detailed list of candidate genes predicted in the QTLs intervals on chromosome 1, 3, and 11 is presented in File S1. The *qRLH1.1* consists of some important genes such as pectin acetyl esterase (LOC_Os01g66830, LOC_Os01g66840, and (LOC_Os01g66850), serine/threonine protein kinase (LOC_Os01g66860), BTBZ1-Bric-a-Brac, Tram track (LOC_Os01g66890), Ser/Thr protein phosphatase (LOC_Os01g66920), zinc finger (LOC_Os01g66970), auxin-responsive protein (LOC_Os01g67030), few expressed protein and transposon proteins. The *qRLH1.2* consists of LTPL39—Protease inhibitor/seed storage/LTP family protein (LOC_Os01g68589). The *qRLH1.5* constitutes NAM protein (no apical meristem protein, LOC_Os01g66490), phosphoribosyl-formyl-glycinamidine synthase (LOC_Os01g66500), MLO domain-containing protein (LOC_Os01g66510), serine/threonine-protein kinase RIO-like (LOC_Os01g66520), C2H2 zinc finger protein (LOC_Os01g66570), and DUF260 domain-containing protein (LOC_Os01g66590). The QTL *qRLH1.8* contains disease resistance-responsive family protein (LOC_01g62030), serine/threonine-protein kinase (LOC_01g62080), thaumatin protein (LOC_01g62260), laccase precursor protein (LOC_01g62480, LOC_01g62490, and LOC_01g62600), aspartic proteinase nepenthesin precursor (LOC_01g62630), and avr9/Cf-9 rapidly elicited protein (LOC_01g62670). A concise list of important disease-resistance candidate genes is given in Table 2. 

## 4. Discussion

### 4.1. O. nivara as a Potential Donor for Resistance and Productivity Traits 

*O. nivara* is a close relative of cultivated rice, sharing the AA genome. Gene transfer from *O. nivara* to cultivated rice is feasible without any major fertility barriers. However, *O. nivara* shows domestication syndrome effects, such as sterile spikelets, long awns, hull color, panicle branching, plant architecture, genetic loci distortion, and high shattering [39,40,41]. In this study, we reported the ShB resistance QTLs from partially resistant wild rice accession of *O. nivara* IRGC81941A. Wild rice *O. nivara* has been proven as a rich reservoir of several important resistance and productivity traits. Our laboratory is maintaining approximately 400 accessions of *O. nivara* and screened for different pathogens and insect pest resistance including sheath blight [32], bacterial blight [42,43], brown planthopper [30,44,45], and productivity traits [46]. It is noteworthy to mention that ShB resistance QTLs were mapped earlier from two *O. nivara* accessions [47]. Additionally, traits such as purple sheath color, plant height, seedling vigor, and yield were also identified and mapped from *O. nivara* [48,49,50,51,52,53,54]. These germplasm resources are potential sources of productivity and resistance traits and offer insights into the domestication history of cultivated rice.

### 4.2. ShB Resistance QTLs 

In this study, we have identified a total of 29 QTLs for ShB resistance using an SNP-based linkage map. Chromosome 1 represents the largest cluster of QTLs in this study. Eleven QTLs were mapped on chromosome 1 (Figure S4a,b). Among them, a cluster of seven QTLs was stably detected across different seasons, including three PH QTLs. Recently, a major stable QTL, *qShB-1.1*, was fine-mapped to a ~0.51 Mb region between RM11935 (37.8 Mb) and RM11968 (38.3 Mb) in this region [55]. Our results also showed that the mapping of PH QTL colocalizes with QTL for RLH, conferring resistance to ShB on chromosome 1. PH QTLs spanned the 35.8-37.4 Mb region. Eizenga et al. [44] mapped a PH QTL partially overlapping this region between SSRs RM315 (36.72 Mb) to RM431 (38.8 Mb) and showed colocalization of *qShB1* between SSRs RM431 (38.8 Mb) and RM1361 (39.0 Mb). This region on chromosome 1 might be a hotspot for imparting ShB tolerance.

In addition to chromosome 1, the present study demonstrates the mapping of stable QTL on chromosome 3 that shows a consistent response to sheath blight disease. This genetic locus overlaps with QTL *QSbr3a* [15]. The report also identifies a consistent QTL cluster of four loci (three affecting LH and one affecting RLH) on chromosome 11, coinciding with the previously mapped major locus qSBR11-1 from Tetep [20]. Additionally, it shows the colocalization of another locus, *qSBR7-1*, identified from Tetep on chromosome 7 [20]. Likewise, our study observed the localization of the previously mapped ShB resistance QTL *qsbr2.1* on chromosome 2, marked by the SSR markers RM8252 and RM8254, which is associated with the severity of sheath blight disease affecting RLH [56]. These findings highlight potential regions on the rice chromosomes that can be further exploited for accelerated breeding of ShB disease resistance.

In our study, we observed that phenotypic screening was significantly influenced by environmental factors in the field, which in turn affected the detection and stability of QTLs. In recent years, sudden variations in environmental factors such as temperature, humidity, light intensity, nitrogen fertilizers, and silica levels in soil have significantly altered disease expression, leading to variability in lesion development and RLH measurements [8,25]. Previous studies reported the environmental fluctuations impacted the expression of disease resistance traits which often result in the detection of multiple inconsistent QTL in the same population across different environment. To identify false positive, an effective strategy is to compare the colocalization of QTL with those previously reported ShB resistance QTL [57]. To improve the selection efficiency of these QTL, markers-assisted selection (MAS) could be a viable alternative.

Besides the colocalization of ShB resistance QTLs, many other agronomically important QTLs were observed in the consistent QTL region on chromosomes 1, 3, and 11. Colocalization of QTLs was determined by the searching coordinates of the SNP markers flanked to the ShB QTLs on the QTL Annotation Rice Online database (Q-TARO, http://qtaro.abr.affrc.go.jp/, accessed on 10 July 2024). It is noted that the stable QTL on chromosome 1, flanked by SNP markers rs.Chr1.38678740 and rs.Chr1.39839342 share a genomic region of several QTLs of agronomic importance including panicle morphology, plant height, yield, and tiller number [58,59,60,61,62,63]. It is worth mentioning that the semi-dwarf gene *sd-1* is also localized in this region [64]. Stable QTL on chromosome 3 flanked by SNP markers rs.Chr3.2694761 and rs.Chr3.3801316 overlaps with root, hybrid sterility, leaf development, and yield-related QTLs [60,65,66,67]. Interestingly, the stable QTL region on chromosome 11 coincides with the recombination suppressor region due to paracentric inversion [68]. No other agronomic traits are reported in this region except *qSBR11.1*, *qLH11.1*, *qLH11.2*, *qLH11.3,* and *qRLH11.1.* Different QTLs at the ShB resistance locus mark the presence of a QTL hotspot. Transferring these genomic blocks carrying QTLs for different traits could lead to the development of sheath blight resistant rice cultivars with superior agronomic traits. 

### 4.3. Candidate Genes Confer Resistance to R. solani

With the mapping of the ShB resistance QTL, a cluster of four ShB resistance QTLs *qRLH1.1*, *qRLH1.2*, *qRLH1.5*, and *qRLH1.8* were mapped at higher resolution using SNP markers on chromosome 1. The total size of QTLs mapped in this cluster ranged from 0.096-420.1 kb. Several important candidate genes have been predicted in this cluster such as pectin acetyl esterase (LOC_Os01g66830, LOC_Os01g66840, and LOC_Os01g66850), serine/threonine protein kinase (LOC_Os01g66860), Ser/Thr protein phosphatase (LOC_Os01g66920), zinc finger (LOC_Os01g66970), auxin-responsive protein (LOC_Os01g67030). Ser/Thr protein kinase, and Ser/Thr protein phosphatase have appeared to be important molecular players in plant defense [69,70]. In a recent report, Ser/Thr protein kinase, and Ser/Thr protein phosphatase have been reported to play an important role in ShB resistance [55]. Protein kinase domain-containing protein has also been shown to regulate the ShB resistance, positively without any substantial trade-offs between resistance and plant growth [29]. The *qRLH1.2* consists of LTPL39—Protease inhibitor/seed storage/LTP family protein (LOC_Os01g68589). Recently, a genome-wide study revealed the role of protease inhibitor/seed storage/LTP family protein in ShB resistance [71]. The *qRLH1.5* harbors NAM protein (no apical meristem protein, LOC_Os01g66490), phosphoribosyl-formyl-glycinamidine synthase (LOC_Os01g66500), MLO domain-containing protein (LOC_Os01g66510), serine/threonine-protein kinase RIO-like (LOC_Os01g66520), C2H2 zinc finger protein (LOC_Os01g66570), and DUF260 domain-containing protein (LOC_Os01g66590). The role of the MLO domain-containing protein, serine/threonine-protein kinase, and C2H2 zinc finger protein has been reported in many fungal and bacterial diseases [69,72]. The QTL *qRLH1.8* contains disease resistance-responsive family protein (LOC_01g62030), serine/threonine-protein kinase (LOC_01g62080), thaumatin protein (LOC_01g62260), laccase precursor protein (LOC_01g62480, LOC_01g62490, and LOC_01g62600), aspartic proteinase nepenthesin precursor (LOC_01g62630), and avr9/Cf-9 rapidly elicited protein (LOC_01g62670). The function of a thaumatin-like protein (TLP) has been demonstrated successfully in the management of sheath blight in rice by over-expression of TLP genes [73,74]. The aspartic proteinase nepenthesin was shown to be significantly linked with ShB resistance in rice [71]. However, quantitative resistance conferred by the resistance gene against sheath blight is still unclear. Therefore, we cannot overlook the possibility of the involvement of the other candidate genes. Many more efforts are needed to confirm the role of these genes in ShB resistance in wild rice *O. nivara.* Further, characterization of the candidate genes will pave a new path to understanding the mechanism of resistance conferred by these genes. 

### 4.4. Breeding Applications for Developing ShB-Resistant Rice Cultivars 

Only a limited number of partially resistant sources have been identified for addressing sheath blight disease in rice. Partial resistance is determined by quantitative traits, and its expression is highly influenced by the environment, making it difficult to consistently detect the associated QTLs across multiple years of screening. While some loci have been precisely mapped, the majority of QTLs are still in the early mapping stage, spanning extensive genomic regions. QTLs with larger genomic regions pose challenges for transfer, as recombination events between genes and markers can impede successful transfer of the QTLs. Furthermore, breeding ShB-resistant cultivars is exceptionally challenging due to the scarcity of effective resistance genes and the emergence of newly evolved resistance pathogens [9]. Despite these challenges, attempts have been made to pyramid ShB resistance QTLs for the development of elite ShB-resistant cultivars [75,76,77]. The identification of a new source of partial resistance from wild rice *O. nivara* acc. IRGC81941A will contribute to broadening the genetic diversity of the resistance gene pool for *R. solani*. The advanced backcross populations generated in this study will serve as valuable breeding materials for improving susceptible cultivars. Notably, three of these populations (1554, 1556, and 1547), which exhibit early maturity and semi-dwarf stature, are currently undergoing initial yield trials for varietal development. Overall, this study enhances our understanding of the underlying QTLs that control resistance to *R. solani*, provides closely linked SNP markers for marker-assisted selection (MAS), and generates pre-breeding materials to develop resilient cultivars capable of withstanding the devastating impacts of sheath blight.

## 5. Conclusions

In conclusion, our study highlights the significant potential of *O. nivara* for enhancing sheath blight resistance in cultivated rice. The consistent disease resistance observed in *O. nivara* acc. IRGC81941A, with a mean RLH of 19–24%, contrasts with the high susceptibility of PR121 (mean RLH of 62–75%). our study demonstrated the mapping of the 29 major and minor QTLs in wild rice (*O. nivara*) for sheath blight resistance. In this study, a cluster of four QTLs (*qRLH1.1*, *qRLH1.2*, *qRLH1.5*, and *qRLH1.8*) on chromosome 1 are stably detected across the multiple seasons. Key markers such as RM212 and RM246, linked to this cluster, have been validated on F5 and a rice diversity panel, showing strong potential for marker-assisted selection (MAS). Interspecific derivatives from *O. nivara* exhibited superior agronomic traits, yield, and sheath blight resistance, making them valuable prebreeding materials for enhancing resistance in cultivated rice varieties.

## Figures and Tables

**Table 1:** Details of QTL mapped for plant height, lesion height, and relative lesion height in the years 2017 and 2018, and their mean.

Seasons	Traits	LGs	QTLs	Left Marker	Right Marker	Genetic Distance (cM)	LOD Score	Additive	R^2^
2017	Plant Height	1	*qPH1.1*	rs.Chr1.37378469	rs.Chr1.37464136	1321.1–1324.9	13.0331	13.923	0.173
		4	*qPH4.1*	rs.Chr4.13005766	rs.Chr4.13217468	1160.8–1164.7	2.9013	1.6064	0.2412
		6	*qPH6.1*	rs.Chr6.12096188	rs.Chr6.11979786	499.3–510.9	2.8491	6.2268	0.0502
		8	*qPH8.1*	rs.Chr8.17265874	rs.Chr8.15842297	487.0–490.9	2.9012	−5.2969	0.1062
	Lesion Height	2	*qLH2.1*	rs.Chr2.35457758	rs.Chr2.35457823	0–2.7	3.3578	−1.9855	0.1602
		3	*qLH3.1*	rs.Chr3.2694761	rs.Chr3.3801316	605.7–626.6	2.7423	−0.8633	0.11
		7	*qLH7.1*	rs.Chr7.17816084	rs.Chr7.17848951	378.5–386.6	3.4374	2.1383	0.1834
		11	*qLH11.1*	rs.Chr11.27787652	rs.Chr11.28970389	31.5–34.8	2.8294	−2.0404	0.2308
		11	*qLH11.2*	rs.Chr11.26522147	rs.Chr11.26053892	90.5–98.5	2.863	1.8248	0.1896
	Relative Lesion Height	1	*qRLH1.1*	rs.Chr1.38966610	rs.Chr1.38764373	1345.9–1353.8	9.9124	−7.0955	0.1667
		1	*qRLH1.2*	rs.Chr1.39839247	rs.Chr1.39839342	1442.4–1443.5	3.3124	0.6929	0.1001
		6	*qRLH6.1*	rs.Chr6.28594729	rs.Chr6.29343276	927.3–932.9	3.4633	1.7665	0.1161
		8	*qRLH8.1*	rs.Chr8.17265874	rs.Chr8.15842297	487.0–490.9	3.4087	3.4381	0.1458
2018	Plant Height	1	*qPH1.2*	rs.Chr1.35874500	rs.Chr1.37378469	1315.6–1321.1	3.1704	12.997	0.2974
		2	*qPH2.1*	rs.Chr2.22628796	rs.Chr2.22747179	624.4–632.5	3.1502	−7.2949	0.0745
	Lesion Height	3	*qLH3.2*	rs.Chr3.2694761	rs.Chr3.3801316	605.7–626.6	4.9772	−1.6642	0.1768
	Relative Lesion Height	1	*qRLH1.3*	rs.Chr1.614777	rs.Chr1.649072	307.7–326.3	4.7678	2.4154	0.4805
		1	*qRLH1.4*	rs.Chr1.649072	rs.Chr1.613037	326.3–338.2	3.7844	2.1927	0.4468
		1	*qRLH1.5*	rs.Chr1.38678740	rs.Chr1.38601901	1364.3–1367.5	11.8044	−9.9129	0.2236
		3	*qRLH3.1*	rs.Chr3.2801043	rs.Chr3.3215296	566.3–573.8	2.672	−3.6511	0.1088
Mean	Plant Height	1	*qPH1.3*	rs.Chr1.35874500	rs.Chr1.37378469	1315.6–1321.1	4.0694	7.5129	0.3769
		2	*qPH2.2*	rs.Chr2.28243124	rs.Chr2.28243186	531.4–532.6	4.0487	−4.5695	0.1224
	Lesion Height	3	*qLH3.2*	rs.Chr3.2801043	rs.Chr3.3215296	566.3–573.8	2.9809	−2.4302	0.1453
		11	*qLH11.3*	rs.Chr11.27721783	rs.Chr11.27721767	43.6–44.1	3.4717	−2.2544	0.0482
	Relative Lesion Height	1	*qRLH1.6*	rs.Chr1.614777	rs.Chr1.649072	307.7–326.3	4.4345	3.3726	0.3992
		1	*qRLH1.7*	rs.Chr1.649072	rs.Chr1.613037	326.3–338.2	3.116	4.4559	0.4052
		1	*qRLH1.8*	rs.Chr1.35880614	rs.Chr1.36300744	1328.8–1336.4	7.1119	−6.4116	0.1372
		2	*qRLH2.1*	rs.Chr2.20104645	rs.Chr2.19443601	762.9–771.0	4.0941	2.9825	0.0595
		11	*qRLH11.1*	rs.Chr11.26611565	rs.Chr11.26522147	83.7–90.5	2.7475	0.6426	0.047

**Table 2:** Candidate genes identified in the genomic region of the locus on chromosome 1.

QTL		Locus Id	Encoded Protein
*qRLH1.1* (202.2 kb)	rs.Chr1.38966610 rs.Chr1.38764373	LOC_Os01g66820	Inactive receptor kinase
		LOC_Os01g66830	Pectinacetylesterase domain protein
		LOC_Os01g66840	Pectinacetylesterase domain protein
		LOC_Os01g66850	Pectinacetylesterase domain protein
		LOC_Os01g66860	Serine/threonine-protein kinase receptor
		LOC_Os01g66890	BTBZ1—Bric-a-Brac, Tramtrack
		LOC_Os01g66920	Ser/Thr protein phosphatase protein
		LOC_Os01g66970	Zinc finger, C3HC4 type domain protein
		LOC_Os01g67030	Auxin-responsive protein
*qRLH1.2* (0.096 kb)	rs.Chr1.39839247 rs.Chr1.39839342	LOC_Os01g68589	LTPL39—Protease inhibitor/seed storage/LTP family protein precursor, expressed
*qRLH1.5* (76.84 kb)	rs.Chr1.38678740 rs.Chr1.38601901	LOC_Os01g66490	No apical meristem protein
		LOC_Os01g66510	MLO domain containing protein
		LOC_Os01g66520	serine/threonine-protein kinase RIO-like
*qRLH1.8* (420.1 kb)	rs.Chr1.35880614 rs.Chr1.36300744	LOC_01g62030	Disease resistance-responsive protein
		LOC_01g62080	Serine/threonine-protein kinase
		LOC_01g62190	ZOS1-15—C2H2 zinc finger protein
		LOC_01g62260	Thaumatin, putative, expressed
		LOC_01g62410	MYB family transcription factor
		LOC_01g62460	ZOS1-16—C2H2 zinc finger protein
		LOC_01g62480	Laccase precursor protein
		LOC_01g62490	Laccase precursor protein
		LOC_01g62510	WRKY119
		LOC_01g62514	WRKY56
		LOC_01g62570	ATP/GTP/Ca++ binding protein
		LOC_01g62584	Peptidase aspartic family protein
		LOC_01g62600	Laccase precursor protein
		LOC_01g62610	Peptidyl-prolyl cis-trans isomerase
		LOC_01g62620	DHHC zinc finger domain protein
		LOC_01g62630	Aspartic proteinase nepenthesin
		LOC_01g62640	Zinc finger, C3HC4 type domain protein
		LOC_01g62650	Pumilio-family RNA binding protein
		LOC_01g62660	MYB family transcription factor
		LOC_01g62670	avr9/Cf-9 rapidly elicited protein

## Data Availability

The original contributions presented in the study are included in the article/Supplementary Materials, further inquiries can be directed to the corresponding author.

## Supplementary Materials

The following supporting information can be downloaded at: https://www.mdpi.com/article/10.3390/genes15070919/s1, Table S1: Descriptive statistics of sheath blight component traits in the F_2_ population; Table S2: Evaluation of 241 F_3_ family (each comprising 10 plants) during the year 2018; Table S3: Promising BC_2_F_3_ lines with consistent resistance response over two years of screening against *R. solani*; Table S4: SNP markers used in mapping of ShB resistance QTL; File S1: Detailed list of candidate genes identified in the stable QTL region; Figure S1: Pearson correlation coefficient of PH, LH, and RLH in the years 2017, 2018 and their mean; Figure S2: Agarose gel representing amplification of SSR marker RM212 and RM246 in some non-basmati, landraces, and basmati rice cultivars; Figure S3: Polymorphic SSR markers of rice chromosome 1, 3 and 11 differentiating the *O*. *nivara* (resistant parent) with PR121(susceptible cultivars); Figure S4: Location of QTL mapped in this study and their comparison with previously mapped ShB resistance QTLs.
