# Supplementary material for: Molecular Mapping and Transfer of Quantitative Trait Loci (QTL) for Sheath Blight Resistance from Wild Rice Oryza nivara to Cultivated Rice (Oryza sativa L.)
Source: Genes (Basel). 2024 Jul 14;15(7):919. doi: 10.3390/genes15070919 (PMC11275441; doi:10.3390/genes15070919)
Supplement: Supplementary file 1 [file genes-15-00919-s001.zip › Table S1.pdf]

Table S1: Descriptive statistics of sheath blight component traits in the F_2 population.

Traits	*O. nivara* IRGC81941A	PR121	Range	Mean	SD	Kurt	Skew	*w* (p-value)
LH	25	54	21.0-52.0	37.8	4.76	3.09	0.16	0.98 (5.71^{-4})
RLH	16	58	0.15-0.70	0.35	0.07	2.53	-0.01	0.95 (3.88^{-10})
PH	155	92	65.0-185.0	110.5	16.80	3.18	-0.14	0.98 (1.40^{-3})

Abbreviations: *LH* Lesion height, *RLH* Relative lesion height, *PH* Plant height, *SD* Standard deviation, *Kurt* Kurtosis, *Skew* Skewness, *w* Shapiro-Wilk statistic value
