# Supplementary material for: Molecular Mapping and Transfer of Quantitative Trait Loci (QTL) for Sheath Blight Resistance from Wild Rice Oryza nivara to Cultivated Rice (Oryza sativa L.)
Source: Genes (Basel). 2024 Jul 14;15(7):919. doi: 10.3390/genes15070919 (PMC11275441; doi:10.3390/genes15070919)
Supplement: Supplementary file 1 [file genes-15-00919-s001.zip › Table S2.pdf]

Table S2: Evaluation of 241 F_3 family (each comprising 10 plants) during the year 2018

Ldh_2018	Mean PH	Mean LH	Mean RLH
PR121	94.1	65.5	69.60
IRGC81941A	155.4	32.3	20.77
14833	141.1	35.3	25.02
14834	129	53.4	41.40
14835	145.1	41.3	28.46
14836	135.5	36.3	26.79
14837	117.3	41.8	35.71
14838	109.8	47.5	43.28
14839	83.4	34.6	41.54
14840	127.9	45.1	35.26
14841	141.8	47.2	33.29
14842	106.1	44.3	41.82
14843	135	48.3	35.80
14844	80.1	46	57.43
14845	119.2	44.7	37.50
14846	101.3	50.5	49.85
14847	117.5	39	33.19
14848	116.4	41.7	35.82
14849	110.7	44.1	39.84
14850	128.8	50	38.82
14851	130.8	37	28.29
14852	121.5	64.1	52.74
14853	123	50.7	41.28
14854	140	47.2	33.71
14855	114.9	46.4	40.38
14856	146.1	43.6	29.84
14857	102.2	36.5	35.77
14858	132.7	44.6	33.61
14859	84	51.3	61.11

14860	86.5	44.9	51.91
14861	119.7	47.5	39.68
14862	113	46.3	41.00
14863	126.8	52.6	41.48
14864	142.7	58.4	40.93
14865	127.6	56.8	44.56
14866	116.7	42.9	36.76
14867	121.7	47	38.62
14868	95	52.2	54.95
14869	110.1	57.1	51.86
14870	77.9	52.3	67.14
14871	129.8	51.6	39.75
14872	114	38.4	33.68
14873	119.4	57.4	48.07
14874	123.4	37.6	30.47
14875	119.1	60.9	51.13
14876	107	57.6	53.83
14877	115.4	57.7	50.00
14878	121.1	65.4	54.00
14879	123.2	51.2	41.56
14880	123.9	55.2	44.55
14881	136.8	60.5	44.23
14882	132.9	62.8	47.25
14883	169.1	64.4	38.08
14884	87	53.7	61.81
14885	108.8	51.8	47.61
14886	97.4	52.4	53.80
14887	155.7	51.7	33.20
14888	148.3	70	47.20
14889	154.7	59	38.14
14890	110	54.5	49.61

14891	94.1	63.2	67.18
14892	-	-	-
14893	143.8	42.6	29.58
14894	112.6	57.4	51.01
14895	98.1	54	55.05
14896	142.7	52	36.44
14897	127.3	45.5	35.74
14898	141.8	47.6	33.57
14899	-	-	-
14900	131.2	46.5	35.48
14901	124.5	38.8	31.16
14902	136.9	55.1	40.25
14903	118.7	46.2	38.92
14904	126.5	42.3	33.45
14905	113	46.9	41.50
14906	145	48	33.10
14907	123.3	47.2	38.28
14908	132.8	52	39.16
14909	143.1	55.2	38.57
14910	139.5	43.8	31.40
14911	-	-	-
14912	121.2	41.9	34.57
14913	142.7	40.3	28.24
14914	85.5	40.1	46.90
14915	117.5	50.1	42.64
14916	111.1	47.3	42.57
14917	111.8	52.8	47.23
14918	132.5	44.3	33.43
14919	146.1	42.7	29.23
14920	135.1	47	34.79
14921	115.3	50.7	43.97

14922	94.2	45.7	48.51
14923	111.5	54.8	49.15
14924	140.9	47.1	33.43
14925	134.6	49.5	36.78
14926	141.6	55.2	39.01
14927	122.5	56.1	45.80
14928	144.1	58.3	40.46
14929	123	47.1	38.29
14930	118.1	49.5	41.96
14931	99	56.8	57.37
14932	108.1	25	23.13
14933	132.4	43.6	32.93
14934	145.3	44.2	30.42
14935	93	39.3	42.26
14936	117.6	45.9	39.03
14937	87.5	48.75	55.71
14938	99	47.7	48.18
14939	76.3	52.5	68.81
14940	134.2	50.1	37.33
14941	89.1	56.8	63.84
14942	107.4	48.2	44.88
14943	131.7	62.2	47.23
14944	124.9	58.2	46.60
14945	-	-	-
14946	146.4	52	35.52
14947	75.4	53.7	71.22
14948	117.1	54.2	46.29
14949	138	52.8	38.26
14950	135.4	56.8	41.95
14951	118	52.5	44.49
14952	163	49.4	30.31

14953	106	39.8	37.55
14954	138.9	47.8	34.41
14955	116.1	42.7	36.78
14956	81.4	54.5	66.95
14957	127.9	44.6	34.87
14958	116.5	56.8	48.76
14959	151	48	31.79
14960	129.9	46.5	35.80
14961	129.4	44.2	34.16
14962	112.3	57.7	51.38
14963	128.7	58.6	45.53
14965	114.6	61.3	53.49
14966	131.3	52.1	39.68
14967	115	49.2	42.78
14968	133.4	47.3	35.46
14969	129.5	55.5	42.86
14970	110.2	44.7	40.56
14971	98.9	53.8	54.40
14972	147.2	55.2	37.50
14973	128.8	46.4	36.03
14974	133.3	47.3	35.48
14975	95.7	50.7	52.98
14976	130.3	50.7	38.91
14977	166.5	57.9	34.77
14978	147.4	59.6	40.43
14979	131.6	59.2	44.98
14980	143.5	48.1	33.52
14981	147	48.9	33.27
14982	144.3	52.9	36.66
14983	129.1	56	43.38
14984	155.8	55.3	35.49

14985	136.3	53.7	39.40
14986	137.3	49.7	36.20
14987	145.2	43.1	29.68
14988	123.2	58	47.08
14989	104.4	50.5	48.37
14990	89.8	45.4	50.56
14991	115.5	55.8	48.31
14992	112.3	58.8	52.36
14993	127.9	38.2	29.87
14994	105.8	39	36.86
14995	116.8	34.3	29.37
14996	126.3	35.7	28.27
14997	126.8	31.4	24.76
14998	123.5	41.3	33.44
14999	124.8	38.8	31.09
15000	141.4	45.4	32.11
15001	164	46.8	28.59
15002	125.8	41.7	33.15
15003	112	35.6	31.79
15004	153	44.75	29.25
15005	-	-	-
15006	87.8	38	43.28
15007	137.3	50.8	37.00
15008	87.6	45	51.37
15009	130.4	37.4	28.68
15010	-	-	-
15011	124.6	43.3	34.76
15012	120.6	38.5	31.92
15013	140.8	39.9	28.34
15014	118.3	43.4	36.69
15015	122.5	39.3	32.08

15016	151.1	42.2	27.93
15017	145.1	40.6	27.98
15018	130.9	54.6	41.71
15019	126.7	36.9	29.12
15020	132.3	37.8	28.57
15021	126.3	45.8	36.26
15022	121.2	44.4	36.66
15023	-	-	-
15024	129.4	44.7	34.54
15025	151.3	47.3	31.26
15026	127.6	41.3	32.37
15027	128.22	36.5	28.51
15028	132.7	46	34.66
15029	156.2	51.7	33.14
15030	137.2	48.2	35.13
15031	96.8	50.3	51.95
15032	-	-	-
15033	132.5	68.8	51.97
15034	138.4	49	35.40
15035	131	48.2	36.79
15036	152.4	48.5	31.82
15037	116.9	47.8	40.89
15038	-	-	-
15039	116	50.3	43.36
15040	146.7	38.7	26.38
15041	132.8	53.1	39.98
15042	138.9	47.5	34.20
15043	153.1	35.1	22.93
15044	132.3	42.6	32.20
15045	131.3	52.2	39.76
15046	144.6	51.8	35.87

15047	128.3	53.3	41.54
15048	149.4	47.8	31.99
15049	124	46.4	37.46
15050	89.6	58	64.73
15051	96.3	53.7	55.76
15052	110.3	43.3	39.26
15053	168.4	49	29.10
15054	150.1	52.5	34.98
15055	146.3	39.5	27.00
15056	145.4	44.3	30.47
15057	168	44.9	26.73
15058	134.4	47.4	35.27
15059	127.3	45.6	35.82
15060	139.5	48.1	34.48
15061	114.4	46.3	40.47
15062	124.9	36.5	29.22
15063	94.8	41.7	43.99
15064	117.6	48.1	40.90
15065	129.2	49.7	38.47
15066	70.2	56.4	80.34
15067	159	48.2	30.31
15068	136.6	53.3	39.02
15069	146.2	51.8	35.47
15070	159.4	53.6	33.63
15071	124.8	43.1	34.54
15072	123.2	44.7	36.28
