# Supplementary material for: Molecular Mapping and Transfer of Quantitative Trait Loci (QTL) for Sheath Blight Resistance from Wild Rice Oryza nivara to Cultivated Rice (Oryza sativa L.)
Source: Genes (Basel). 2024 Jul 14;15(7):919. doi: 10.3390/genes15070919 (PMC11275441; doi:10.3390/genes15070919)
Supplement: Supplementary file 1 [file genes-15-00919-s001.zip › Table S3.pdf]

Table S3 Promising BC_2F_3 lines with consistent resistance response over two years of screening against *R. solani*

Line number	Pedigree	2019				2020		
		Mean PH (cm)	Mean LH (cm)	RLH (%)	DF	Mean PH (cm)	Mean LH (cm)	RLH (%)
**BC_2F_3 [PR114/ *O. nivara* IR81941A]//2*PR114**								
PR114	-	98	69	70.4	135	99	65	65.6
PR121	-	97	63.6	65.5	140	96	64.5	64.5
O. nivara	-	160.4	22.5	14.0	155	163.3	25.5	15.6
1554-7	2k15-1341-1-1-6-7	82.0	20.6	25.1	130	90.0	20.0	22.2
1554-33	2k15-1341-1-1-6-34	88.0	24.4	27.7	121.0	98.0	21.0	21.4
1554-37	2k15-1341-1-1-6-37	96.0	20.4	21.3	111.0	105.0	21.7	20.6
1554-38	2k15-1341-1-1-6-38	84.0	19.8	23.6	108.0	94.7	20.0	21.1
1554-43	2k15-1341-1-1-6-43	82.0	18.8	22.9	115.0	95.0	20.0	21.1
1554-62	2k15-1341-1-1-6-62	89.0	13.0	14.6	121.0	94.0	20.0	21.3
1554-65	2k15-1341-1-1-6-65	87.0	23.6	27.1	111.0	100.7	23.3	23.2
1554-72	2k15-1341-1-1-6-72	86.0	23.8	27.7	108.0	91.0	21.7	23.8
1554-75	2k15-1341-1-1-6-75	91.0	24.8	27.3	108.0	91.0	21.7	23.8
1554-81	2k15-1341-1-1-6-81	96.0	21.8	22.7	103.0	94.0	23.3	24.8
1554-86	2k15-1341-1-1-6-86	81.0	22.8	28.1	96.0	90.0	22.3	24.8
1554-93	2k15-1341-1-1-6-93	82.0	22.6	27.6	96.0	90.0	22.7	25.2
1554-101	2k15-1341-1-1-6-101	85.0	20.2	23.8	106.0	89.0	25.0	28.1
**BC_2F_3 [PR114/ *O. nivara* IR81941A]//2*PR114**								
1556-1	2k15-1341-1-1-13-1	90	18.4	20.44	134	85.0	23.3	27.5
1556-2	2k15-1341-1-1-13-2	92	14.4	15.65	120	90.0	20.0	22.2
1556-3	2k15-1341-1-1-13-3	82	21.2	25.85	124	80.0	20.0	25.0
1556-4	2k15-1341-1-1-13-4	85	23.6	27.65	130	85.0	20.0	23.5
1556-11	2k15-1341-1-1-13-11	91	22.6	24.84	120	90.0	33.3	37.0
1556-16	2k15-1341-1-1-13-16	72.0	17.6	24.4	111	80.0	23.3	29.2
1556-42	2k15-1341-1-1-13-42	102.0	25.4	24.9	120	100.0	23.3	23.3
1556-43	2k15-1341-1-1-13-43	100.0	22.6	22.6	110	95.0	20.0	21.1
1556-44	2k15-1341-1-1-13-44	98.0	24.0	24.5	115	85.0	25.0	29.4
1556-45	2k15-1341-1-1-13-45	96.0	19.2	20.0	112	75.0	25.0	33.3
1556-49	2k15-1341-1-1-13-49	85.0	24.6	28.9	124	85.0	20.0	23.5

1556-50	2k15-1341-1-1-13-50	87.0	22.4	25.7	123	90.0	18.3	20.4
1556-51	2k15-1341-1-1-13-51	79.0	18.4	23.3	121	85.0	25.0	29.4
1556-52	2k15-1341-1-1-13-52	74.0	27.0	36.5	120	80.0	25.0	31.3
1556-53	2k15-1341-1-1-13-53	74.0	21.2	28.6	110	90.0	21.7	24.1
1556-55	2k15-1341-1-1-13-55	74.0	31.0	41.9	124	90.0	31.7	35.2
1556-71	2k15-1341-1-1-13-71	80.0	22.2	27.8	110	85.0	23.3	27.5
1556-75	2k15-1341-1-1-13-75	75.0	19.8	26.4	112	70.0	16.7	23.8
1556-76	2k15-1341-1-1-13-76	77.0	22.8	29.6	120	80.0	16.7	20.8
1556-78	2k15-1341-1-1-13-78	84.0	22.6	26.9	113	90.0	25.0	27.8
1556-79	2k15-1341-1-1-13-79	72.0	16.2	22.5	124	90.0	21.7	24.1
1556-80	2k15-1341-1-1-13-80	83.0	17.2	20.7	136	85.0	16.7	19.6
1556-81	2k15-1341-1-1-13-81	84.0	23.8	28.3	136	90.0	16.7	18.5
1556-88	2k15-1341-1-1-13-88	82.0	17.0	20.7	130	95.0	23.3	24.6
1556-89	2k15-1341-1-1-13-89	79.0	23.4	29.6	122	100.0	21.7	21.7
1556-90	2k15-1341-1-1-13-90	80.0	20.4	25.5	111	95.0	21.7	22.8
1556-91	2k15-1341-1-1-13-91	77.0	21.6	28.1	123	75.0	21.7	28.9
1556-100	2k15-1341-1-1-13-100	88.0	22.4	25.5	134	85.0	20.0	23.5
1556-101	2k15-1341-1-1-13-101	84.0	17.0	20.2	137	80.0	20.0	25.0
1556-110	2k15-1341-1-1-13-110	84.0	24.8	29.5	110	105.0	21.7	20.6
**BC_2F_3 [(PR121/*O. nivara* IR81941A)//2*PR121]**								
1547-14	2k15-1350-3-1-37-14	90.0	31.8	35.3	113.0	88.0	20.7	23.5
1547-26	2k15-1350-3-1-37-26	82.0	10.6	12.9	108.0	90.0	22.0	24.4
1547-30	2k15-1350-3-1-37-30	92.0	9.4	10.2	113.0	96.7	28.7	29.7
1547-36	2k15-1350-3-1-37-36	91.0	29.0	31.9	106.0	91.0	24.0	26.4
1547-51	2k15-1350-3-1-37-51	83.0	34.6	41.7	108.0	77.3	24.7	31.9
1547-73	2k15-1350-3-1-37-73	80.0	33.8	42.3	106.0	84.3	24.0	28.5
