# Supplementary material for: Molecular Mapping and Transfer of Quantitative Trait Loci (QTL) for Sheath Blight Resistance from Wild Rice Oryza nivara to Cultivated Rice (Oryza sativa L.)
Source: Genes (Basel). 2024 Jul 14;15(7):919. doi: 10.3390/genes15070919 (PMC11275441; doi:10.3390/genes15070919)
Supplement: Supplementary file 1 [file genes-15-00919-s001.zip › Table S4.pdf]

Table S4: SNP markers used in mapping of ShB resistance QTL

Linkage Number	Total of SNPs	Total no. of polymorphic	Polymorphic markers placed in LGs
Chr01	40,710	6516	289
Chr02	35630	6021	235
Chr03	37276	5143	96
Chr04	30754	4968	242
Chr05	25943	4126	87
Chr06	27631	4908	194
Chr07	23104	3248	114
Chr08	25342	4240	147
Chr09	19636	3617	283
Chr10	18608	3039	141
Chr11	25471	3972	77
Chr12	22558	1522	66
Total	3,32,663	51,320	1,971
