# Supplementary figures and images for: Molecular Mapping and Transfer of Quantitative Trait Loci (QTL) for Sheath Blight Resistance from Wild Rice Oryza nivara to Cultivated Rice (Oryza sativa L.)
Source: Genes (Basel). 2024 Jul 14;15(7):919. doi: 10.3390/genes15070919 (PMC11275441; doi:10.3390/genes15070919)

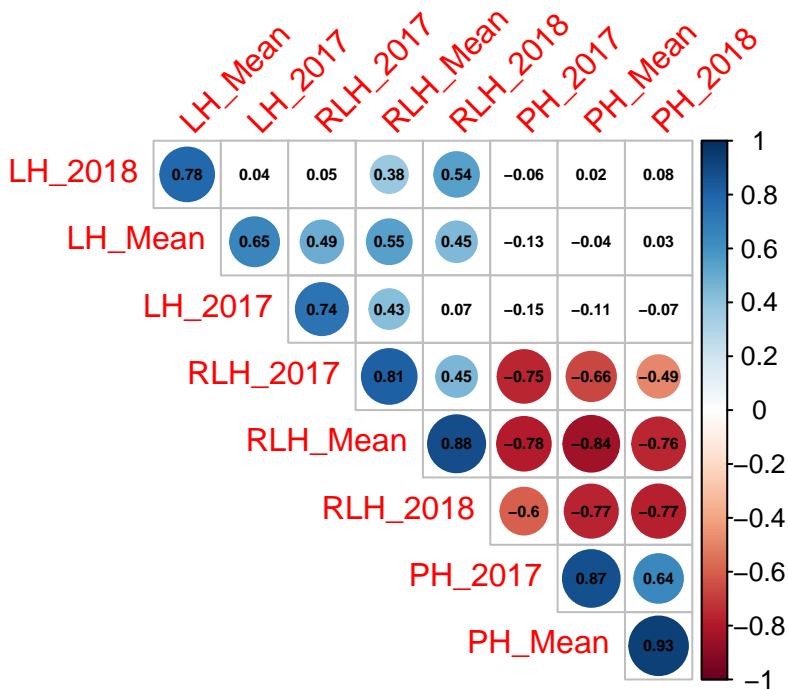

Supplement: Supplementary file 1 [file genes-15-00919-s001.zip › Figure S1.pdf]

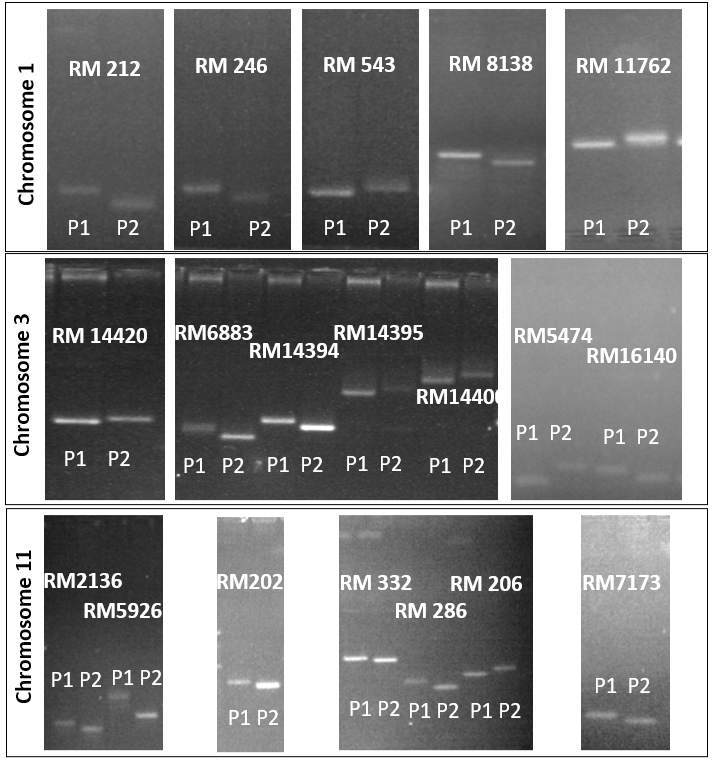

Supplement: Supplementary file 1 [file genes-15-00919-s001.zip › Figure S2.png]

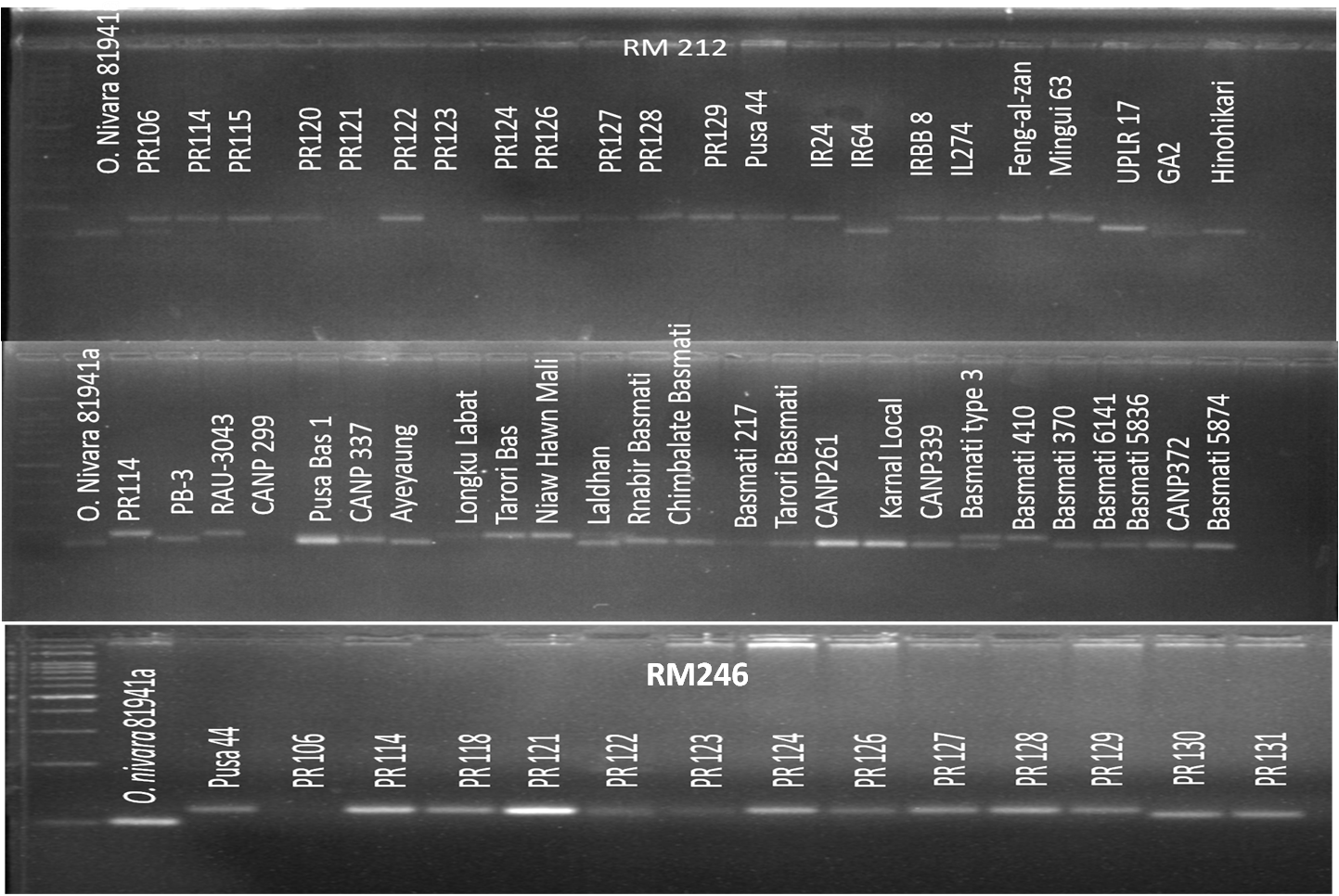

Supplement: Supplementary file 1 [file genes-15-00919-s001.zip › Figure S3.tif]

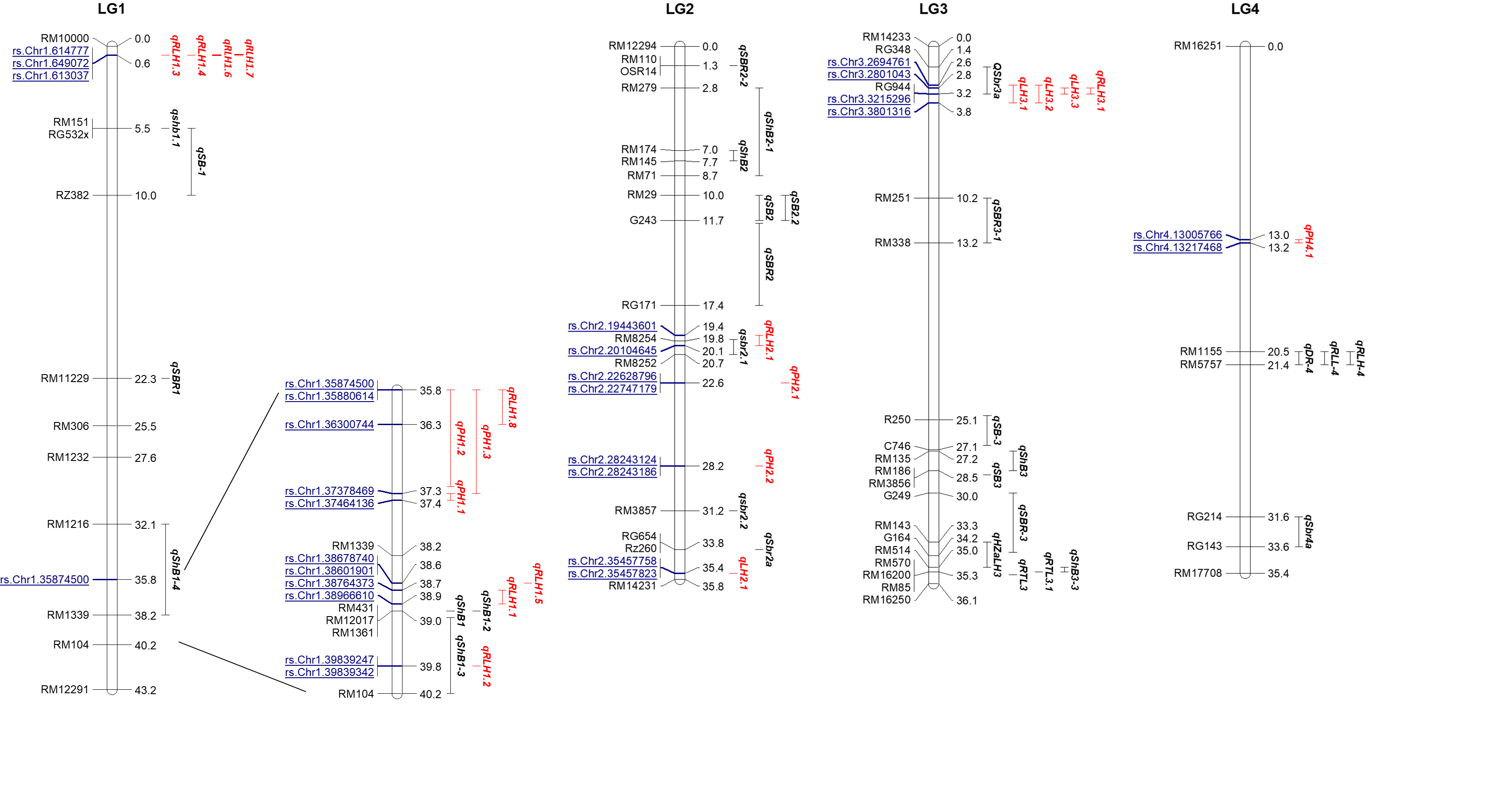

Supplement: Supplementary file 1 [file genes-15-00919-s001.zip › Figure S4a.tif]

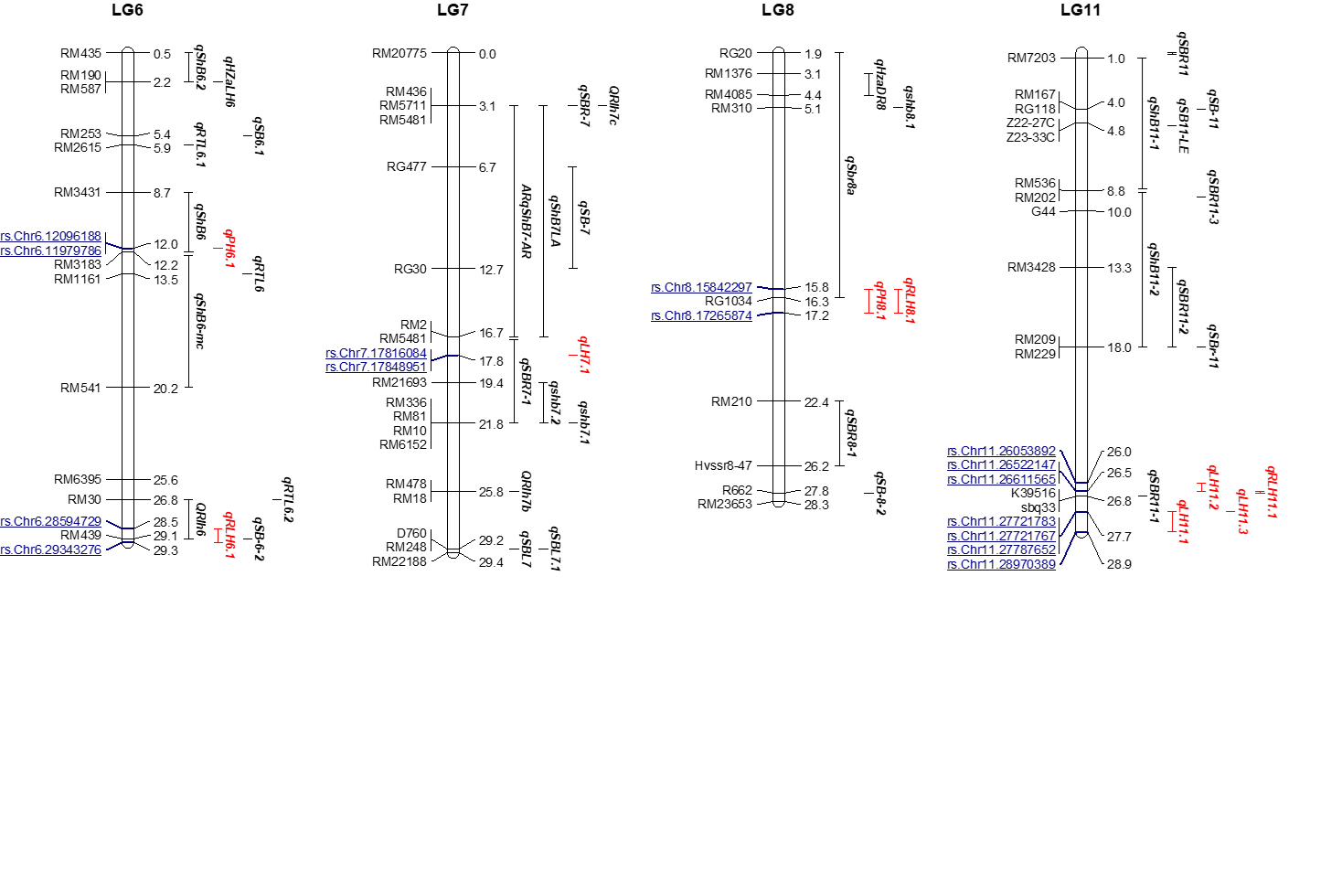

Supplement: Supplementary file 1 [file genes-15-00919-s001.zip › Figure S4b.tif]
